# Recent Update on the Pharmacological Effects and Mechanisms of Dihydromyricetin

**DOI:** 10.3389/fphar.2018.01204

**Published:** 2018-10-25

**Authors:** Jingyao Zhang, Yun Chen, Huiqin Luo, Linlin Sun, Mengting Xu, Jin Yu, Qigang Zhou, Guoliang Meng, Shengju Yang

**Affiliations:** ^1^Department of Dermatology, Affiliated Hospital of Nantong University, Nantong, China; ^2^Department of Pharmacology, School of Pharmacy, Key Laboratory of Inflammation and Molecular Drug Target of Jiangsu Province, Nantong University, Nantong, China; ^3^Department of Pharmacology, School of Pharmacy, Nanjing Medical University, Nanjing, China

**Keywords:** dihydromyricetin, cardioprotection, hepatoprotection, neuroprotection, oxidative stress, apoptosis

## Abstract

As the most abundant natural flavonoid in rattan tea, dihydromyricetin (DMY) has shown a wide range of pharmacological effects. In addition to the general characteristics of flavonoids, DMY has the effects of cardioprotection, anti-diabetes, hepatoprotection, neuroprotection, anti-tumor, and dermatoprotection. DMY was also applied for the treatment of bacterial infection, osteoporosis, asthma, kidney injury, nephrotoxicity and so on. These effects to some extent enrich the understanding about the role of DMY in disease prevention and therapy. However, to date, we still have no outlined knowledge about the detailed mechanism of DMY, which might be related to anti-oxidation and anti-inflammation. And the detailed mechanisms may be associated with several different molecules involved in cellular apoptosis, oxidative stress, and inflammation, such as AMP-activated protein kinase (AMPK), mitogen-activated protein kinase (MAPK), protein kinase B (Akt), nuclear factor-κB (NF-κB), nuclear factor E2-related factor 2 (Nrf2), ATP-binding cassette transporter A1 (ABCA1), peroxisome proliferator-activated receptor-γ (PPARγ) and so on. Here, we summarized the current pharmacological developments of DMY as well as possible mechanisms, aiming to push the understanding about the protective role of DMY as well as its preclinical assessment of novel application.

## Introduction

**FIGURE 1:**
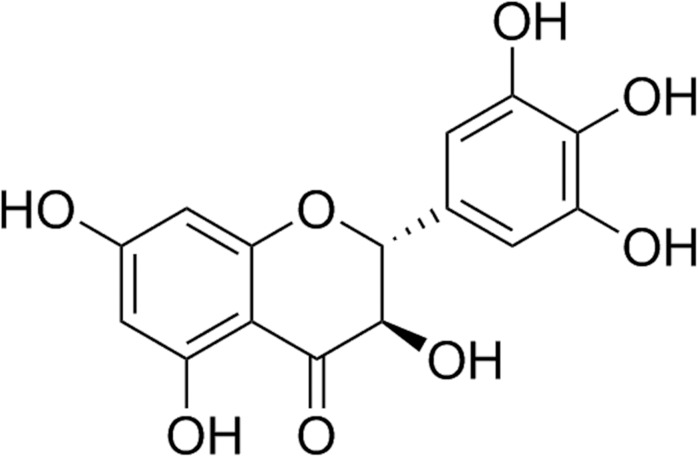
Chemical structure of dihydromyricetin (DMY).

*Ampelopsis grossedentata* (Hand.-Mazz.) W.T. Wang (Vitaceae) is a flavonoid-rich wild plant, which is traditionally used as tea to treat pyretic fever or cough. Its tender stems and leaves are widely used as Vine tea. It has been used for herbal tea and Traditional Chinese Medicine for over hundreds of years. The phytochemical studies showed that dihydromyricetin (DMY, the structure is shown in Figure 1) and myricetin are the two main flavonoids in *A. grossedentata*, respectively. Moreover, the content of DMY (20–30%, w/w) was much more than that of myricetin (1.5–3.0%, w/w).

**FIGURE 2:**
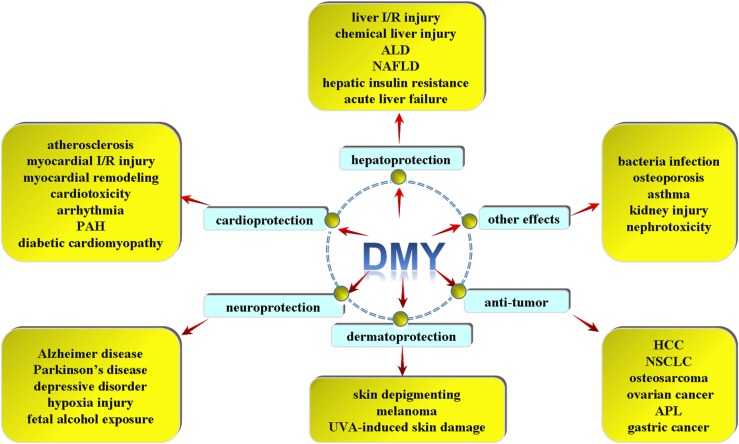
Different pharmacological effects of DMY. DMY exhibits powerful cardioprotection on atherosclerosis, myocardial ischemia reperfusion (I/R) injury, myocardial remodeling, adriamycin-induced cardiotoxicity, arrhythmia, pulmonary artery hypertension (PAH) and diabetic cardiomyopathy (DCM). DMY protects against liver I/R injury, chemical liver injury, alcoholic liver disease (ALD), non-alcoholic fatty liver disease (NAFLD), hepatic insulin resistance and acute liver failure. DMY has been shown to protect against Alzheimer disease (AD), Parkinson’s disease (PD), depressive disorder, hypoxia injury and fetal alcohol exposure induced brain injury. DMY is also indicated to suppress hepatocellular carcinoma (HCC), non-small cell lung cancer (NSCLC), osteosarcoma, ovarian cancer, acute promyelocytic leukemia (APL) and gastric cancer. DMY is beneficial for skin depigmenting, melanoma and UVA-induced skin damage. And DMY was applied for the treatment of anti-bacterial infection, osteoporosis, asthma, kidney injury, nephrotoxicity and so on.

To our knowledge, flavonoids have many peculiar effects such as scavenging oxygen free radicals, anti-oxidation, anti-thrombosis, anti-inflammation, anti-tumor and so on (Jia et al., 2012; de Queiroz et al., 2014; Yang et al., 2015). In addition to the general characteristics of flavonoids, DMY is capable of alleviating inflammation, attenuating lipid consummation, enhancing cholesterol efflux and inhibiting foam cell formation during atherosclerosis. Moreover, DMY exhibits powerful cardioprotection on myocardial ischemia reperfusion (I/R) injury, adriamycin-induced cardiotoxicity, arrhythmia, myocardial remodeling and pulmonary artery hypertension. DMY also reduces blood glucose, improves insulin resistance and attenuates diabetic cardiomyopathy in diabetes. DMY protects liver I/R injury, chemical liver injury, alcoholic liver disease, non-alcoholic fatty liver disease (NAFLD) and acute liver failure (ALF). Besides, DMY has been shown to protect against Alzheimer disease, Parkinson’s disease, depressive disorder, hypobaric hypoxia or fetal alcohol exposure (FAE) induced brain injury and behavioral deficits. DMY is also indicated to suppress hepatocellular carcinoma, non-small cell lung cancer (NSCLC), osteosarcoma, ovarian cancer, melanoma and gastric cancer. DMY also effectively inhibits tyrosinase activity and reduces melanin amount in cells, attenuates melanoma growth, and protects against ultraviolet A-induced skin damage. DMY is also applied for the treatment of bacterial infection, osteoporosis, asthma, kidney injury, nephrotoxicity and so on (Figure 2).

This review will summarize the pharmacological effects and possible mechanisms of DMY.

## Cardiovascular System

### Anti-atherosclerosis

Atherosclerosis is a progressive inflammatory disease caused by fat deposition, thrombus formation, connective tissue proliferation, and calcium carbonate accumulation in blood vessels, which damages vascular endothelium to induce or aggravate the occurrence of vascular diseases (Tang K. et al., 2018; Tang L. et al., 2018).

Various changes during atherosclerosis are commonly considered as different stages of chronic inflammation, suggesting that enhanced inflammation is a critical mechanism in atherosclerosis. Palmitic acid (PA) promoted pyroptosis and pro-inflammatory programmed cell death in human umbilical vein endothelial cells (HUVECs) depending on mitochondrial reactive oxygen species (ROS). The latest research found that DMY (0.5 and 1 μM) pre-treatment for 12 h followed by PA stimulation for another 24 h increased cell viability, decreased lactate dehydrogenase (LDH) and interleukin-1β (IL-1β) release, improved cell membrane integrity, inhibited caspase-1 cleavage and subsequent IL-1β maturation, and finally suppressed ROS generation, which suggested that DMY attenuated PA-induced pyroptosis in HUVECs. Moreover, above protective effects of DMY were unavailable if nuclear factor E2-related factor 2 (Nrf2) was knockdown by Nrf2 siRNA (Hu et al., 2018). The anti-pyrotosis effect on HUVECs suggested a novel mechanism of DMY against inflammation during atherosclerosis.

There are many potential factors involved in elevating inflammatory response and accelerating atherosclerosis. Modified low-density lipoprotein (LDL) especially oxidized LDL (ox-LDL) increases lipids accumulation in macrophages. Enhanced lipid absorbtion as well as impaired cholesterol efflux results in excessive lipoprotein accumulation, foam cells formation, and serious inflammation. In human acute monocytic leukemia cells (THP-1 cells)-derived macrophages, pre-treatment with DMY (1–100 μM) inhibited intracellular cholesterol and lipid accumulation after ox-LDL stimulation. However, DMY had no obvious influence on expressions of scavenger receptor Class A (SR-A) and cluster of differentiation 36 (CD36) (two vital scavenger receptors for cholesterol uptake). Moreover, ATP-binding cassette transporter A1 (ABCA1), ATP-binding cassette transporter G1 (ABCG1) and scavenger receptor-B1 (SR-B1) responsible for cholesterol efflux were increased by DMY in macrophages with or without ox-LDL administration. It is noted that the DMY-mediated cholesterol efflux and suppressive effect on cholesterol accumulation was attenuated after ABCA1 or ABCG1 knockdown. The researchers also confirmed that the enhanced expressions of ABCA1 and ABCG1 by DMY were mediated by liver X receptor α (LXRα) but not peroxisome proliferator-activated receptor-γ (PPARγ). Experiments *in vivo* also found that DMY attenuated the plaque lesion in aortic root, increased LXRα, ABCA1 and ABCG1 expressions in aorta of the apolipoprotein E (apoE)^−/−^ mice with high fat diet (HFD) (Zeng Y. et al., 2018). The reduced lipid absorbtion but enhanced cholesterol efflux by DMY proposed a great possibility for the prevention or treatment for atherosclerosis.

Another study found that DMY also ameliorated hyperlipidemia and inhibited inflammation by reducing serum interleukin-6 (IL-6), tumor necrosis factor-α (TNF-α) mRNA expression, suppressing ROS generation and nicotinamide adenine dinucleotide phosphate (NADPH) oxidase 2 (NOX2), nuclear factor-κB (NF-κB), intercellular adhesion molecule 1 (ICAM-1), vascular cell adhesion molecule-1 (VCAM-1) protein expression in aorta of LDLr^−/−^ mice with HFD. *In vitro*, DMY alleviated monocytes adhesion and attenuated oxidative stress in ox-LDL stimulated HUVECs. In addition, DMY enhanced cholesterol efflux, reduced ox-LDL levels and inhibited foam cell formation (Liu et al., 2017). It is suggested that DMY exerted the anti-atherosclerotic effect through various mechanisms.

Dihydromyricetin also weakened the phosphorylation and degradation of the inhibitor of NF-κB alpha (IκBα), and reduced p65 translocation into the nucleus in TNF-α-induced HeLa cells, which might be related to suppress the upstream signaling of IκB kinase (IKK) through the inhibition of expression of adaptor proteins, TNF receptor-associated factor 2 (TRAF2), and receptor-interacting protein 1 (RIP1). Furthermore, DMY inhibited target genes expressions involved in inflammation and proliferation followed by NF-κB inhibition, which suggested that DMY was a potent natural NF-κB inhibitor (Tang et al., 2016). All those studies verified the potential protective effect against foam cell formation, monocytes adhesion, inflammation and oxidative stress of DMY, which implied that DMY should be a novel therapeutic agent for atherosclerosis.

### Cardioprotection

Myocardial I/R injury refers to the restoration of blood within a certain period of time after myocardial blood supply interruption. Ischemic myocardium damages more severely or undergoes more serious dysfunction, and even with arrhythmia and enlarged infarct size after blood reperfusion (Ravindran et al., 2017). DMY pre-treatment for 7 days attenuated myocardial I/R injury by left anterior descending coronary artery (LAD) ligation for 30 min followed by reperfusion for 4 h *in vivo*. DMY pre-treatment for 24 h also alleviated cell damage by hypoxia for 6 h and reoxygenation for 24 h in H9c2 cardiomyocytes. DMY enhanced antioxidant capacity, inhibited apoptosis, increased phosphatidylinositol 3-kinase (PI3K)/protein kinase B (Akt) but decreased hypoxia-inducible factor -1α (HIF-1α) expression both *in vivo* and *in vitro* (Liu S. et al., 2016). Moreover, the cardioprotective effect of DMY against I/R injury and apoptosis was abolished if PI3K inhibitor LY294002 was pre-administrated, which suggested that PI3K was involved in the protective effect of DMY on myocardial I/R injury.

Adriamycin (ADR) is an effective cytotoxic drug belonging to anthracyclines for oncology. However, ADR has strong cardiotoxicity to induce myocardial cells apoptosis (Praga et al., 1979). One study suggested that DMY (125–500 mg/kg) increased survival rate, improved electrocardiographic disorders, decreased the LDH, alanine aminotransferase (ALT) and creatine kinase MB (CK-MB) levels in serum from imprinting control region (ICR) mice after ADR (20 mg/kg) administration. DMY (50 μM) pre-treatment for 24 h also suppressed apoptosis and attenuated ROS in ADR (2 μM) stimulated neonatal rat cardiomyocytes for another 24 h. Moreover, DMY restored the descending expression of anti-apoptosis protein apoptosis repressor with caspase recruitment domain (ARC), which was related to the inhibitory effect on murine double minute 2 (MDM2) as an E3 ubiquitin ligase of ARC (Zhu et al., 2015). The data indicated that combined usage of DMY was beneficial to attenuating the toxicity of adriamycin on the heart.

Arrhythmia is a common symptom of many heart diseases, characterized by acute onset and high mortality. Studies showed that DMY reduced incidence of aconitine-induced experimental arrhythmias, shortened the action potential duration and decreased amplitude of action potential. The detailed electrophysiological mechanism of DMY is to inhibit sodium currents (*I*_Na_) and to enhance calcium current (*I*_K1_) (Wang et al., 2014). The present results highlight a novel role of DMY against arrhythmia.

Cardiac remodeling, respected as myocardial hypertrophy and myocardial fibrosis, is a compensatory response of the heart to biomechanical stretching and neurohumoral stimulation (Liao R.J. et al., 2014; Woo et al., 2015; Zhao et al., 2017b; Wang et al., 2018). Myocardial remodeling is commonly regarded as an independent risk factor of cardiovascular diseases, which may develop into heart failure and even sudden cardiac death (Meng et al., 2016, 2018). It is important to find out more effective agents to attenuate myocardial hypertrophy. Our present study found that DMY (20, 40, 80, and 160 μM) pre-administration decreased cell areas, inhibited hypertrophic genes expression, attenuated oxidative stress, enhanced cyclic guanosine monophosphate (cGMP) level and endothelial nitric oxide synthase (NOS) phosphorylation at serine 1177 in angiotensin II (Ang II) induced cardiomyocyte hypertrophy. Moreover, the preventive effect of DMY on cardiac hypertrophy was abolished if non-specific NOS inhibitor L-nitro arginine methyl ester (L-NAME) was pre-incubated for 30 min (Meng et al., 2015). Our research also verified the anti-fibrotic effect of DMY on Ang II stimulated cardiac fibroblasts. The experiments *in vitro* showed that DMY pre-incubation attenuated the cardiac fibroblasts proliferation, inhibited type I and type III collagen expression, suppressed α-smooth muscle actin (α-SMA) mRNA and protein level, decreased cellular ROS and malondialdehyde (MDA) level but increased total antioxidant capacity (T-AOC) and superoxide dismutase (SOD) activity. In addition, DMY suppressed p22^phox^ and enhanced thioredoxin (Trx) expression in cardiac fibroblasts after Ang II stimulation (Song et al., 2017). Our latest study verified that 4 weeks of DMY (250 mg/kg/day) intragastric administration attenuated transverse aortic constriction (TAC) induced myocardial hypertrophy via oxidative stress inhibition and sirtuin-3 (SIRT3) pathway enhancement (Chen et al., 2018c). Altogether, above data suggested that DMY might be an ideal natural product to combat myocardial remodeling.

Pulmonary artery hypertension (PAH) is a fatal disease characterized by high pulmonary arterial pressure and pulmonary vasculature remodeling. One group found that DMY decreased right ventricular systolic pressure (RVSP), attenuated right ventricular hypertrophy (RVH), alleviated pulmonary arterial remodeling and reduced IL-6 expression in monocrotaline (MCT) induced PAH of rats. Study *in vitro* also found that DMY (100 μM) pre-treatment for 12 h inhibited IL-6-induced human pulmonary arterial smooth muscle cells (HPASMCs) migration. Moreover, both phosphorylation of signal transducer and activator of transcription 3 (STAT3, as the vital downstream signal of IL-6) and matrix metalloproteinase-9 (MMP9, as a critical mediator for migration) were suppressed by DMY in MCT induced PAH of rats and IL-6 stimulated HPASMCs (Li et al., 2017). DMY pre-treatment significantly attenuated hydrogen peroxide (H_2_O_2_)-induced apoptosis and inhibited intracellular ROS over-production in HUVECs. Further research found that DMY prior to H_2_O_2_ stimulation inhibited p53 activation, followed by regulation of B-cell lymphoma 2 (Bcl-2) and Bcl-2-associated X protein (Bax), reduction of cytochrome c release, cleaved caspase-9 and caspase-3 expression, and then suppression of poly (ADP-ribose) polymerase (PARP) cleavage in HUVECs (Hou et al., 2015). These results suggested that DMY is a promising agent for prevention or treatment of cardiovascular diseases.

## Anti-Diabetes

Dihydromyricetin administration for 8 weeks significantly reduced blood glucose and plasma insulin in rats with HFD-induced insulin resistance. Metabolite profiling suggested that DMY regulated 24 metabolic pathways, including glycolysis, gluconcogenesis, trichloroacetic acid (TCA) cycle, purine metabolism, urea cycle and so on. Further studies verified that DMY promoted the phosphorylation of Akt, Akt substrate of 160 kDa (AS160) and AMP-activated protein kinase (AMPK), which is beneficial to facilitating glucose transporter 1 (GLUT1) translocation because AMPK is a sensitive glucose sensor which contributes to increasing insulin-independent glucose uptake and to maintaining glucose homeostasis. DMY also inhibited glycogen synthase-3β (GSK-3β) to delay the development of insulin resistance (Le et al., 2016). Similar results were also exhibited on 3T3-L1 pre-adipocytes. The researchers found that DMY promoted glucose uptake in both adipogenic differentiation of 3T3-L1 cells and dexamethasone-treated differentiated adipocytes (Liu et al., 2018). Above data suggest that DMY might be powerful to attenuate insulin resistance in type 2 diabetes (T2D).

Insulin resistance in skeletal muscle is a vital characteristic during the pathogenesis of T2D. Previous studies found that DMY up-regulated Akt phosphorylation to promote glucose uptake with or without insulin stimulation in differentiated C2C12 myotubes. Insulin tolerance test indicated that DMY of 50 mg/kg/day for 12 weeks also improved insulin sensitivity in mice. Moreover, further experiments showed that above improved skeletal muscle insulin sensitivity was unavailable in the presence of 3-methyladenine (3-MA), bafilomycin A1 (Baf-A1), or autophagy-related gene 5 (Atg5) siRNA in C2C12 myotubes, which suggested that autophagy enhancement was important for the improved insulin sensitivity by DMY. The studies also found that DMY increased AMPK phosphorylation, elevated peroxisome proliferator-activated receptor coactivator-1a (PGC-1α) expression, and enhanced SIRT3 activation in skeletal muscle. Moreover, PGC-1α or AMPK siRNA transfection abolished DMY-elicited SIRT3 up-regulation and autophagy in C2C12 myotubes (Shi et al., 2015). Altogether, DMY was beneficial to improving skeletal muscle insulin sensitivity in diabetic, and AMPK-PGC-1α-SIRT3 signaling pathway was possible involved in the protective effect.

Another research confirmed that DMY improved insulin sensitivity induced by dexamethasone in adipocytes. They found that DMY increased glucose uptake and decreased adipogenesis in dexamethasone-treated adipocytes. DMY inhibited phosphorylation of PPARγ at serine 273 (Ser273) and suppressed extracellular signal-regulated kinase 1/2 (ERK)/cyclin dependent kinase-5 (CDK5) activity. It is noted that PPARγ inhibitor GW9662 pre-treatment alleviated the beneficial effects of DMY in differentiated adipocytes. Further studies found that combination of DMY with mitogen-activated protein kinase (MEK) inhibitor PD98059 synergistically and robustly improved glucose uptake and adiponectin secretion in adipocytes (Liu et al., 2018). These findings verified the regulatory effect of DMY on insulin resistance, which was critical to essentially alleviate the progress of diabetes.

Diabetes obviously promoted a functional and structural impairment defined as diabetic cardiomyopathy (DCM), which increases the risk of heart failure even in the absence of hypertension and coronary heart disease. One group showed that treatment with DMY for 14 weeks normalized body weight, alleviated pathological changes in the myocardium and improved cardiac function in streptozotocin (STZ)-administrated male C57BL/6 mice. DMY significantly increased the activities of myocardial anti-oxidative enzymes SOD and glutathione peroxidase (GSH-Px), while decreased the level of MDA. DMY also exerted anti-inflammatory effect by reducing IL-6 and TNF-α levels. DMY ameliorated mitochondrial dysfunction through enhancement of ATP content, citrate synthase activity and complex I/II/III/IV/V level. DMY inhibited cardiac apoptosis but restored autophagy in STZ-induced diabetic mice, which might be related to the increased AMPK phosphorylation and downstream target UNC-51 like kinase (ULK1) expression (Wu B. et al., 2017). These results implied that DMY also had a pharmacological effect on diabetic complications.

## Hepatoprotection

The DMY also exhibited protective effects on liver I/R injury. Researches indicated that DMY (100 mg/kg/d) administration daily by gavage 7 days before ischemia obviously decreased serum aminotransferase activity and inhibited apoptosis. The hepatoprotective effects DMY was associated with various essential autophagy-related genes including Atg5, Atg12, beclin 1, and microtubule-associated protein 1 light chain 3 (LC3). Moreover, the improvement of liver function by DMY was attenuated by autophagy inhibitor chloroquine or Atg5 silence. DMY also increased forkhead box O3a (FOXO3a) expression, enhanced Ser588 phosphorylation and promoted FOXO3a nuclear translocation (Chen et al., 2016). These detailed mechanisms were beneficial to promoting the development of DMY-related agents.

Ethanol activates oxygen molecules to produce oxygen free radicals, and leads to lipid peroxidation of hepatocyte membranes, glutathione depletion and MDA accumulation in liver, which are all the accelerators for alcoholic liver disease (ALD). DMY (75 and 150 mg/kg/d) administration for 6 weeks significantly attenuated hepatic enzyme release, alleviated hepatic lipid peroxidation, lessened triglyceride deposition, decreased inflammatory cytokines and partially recovered hepatic pathological injury on C57BL/6 mice fed with Lieber-DeCarli diet containing alcohol. It is noted that DMY ameliorated alcohol-induced glutathione (GSH) depletion and MDA elevation in the liver. The further research verified that DMY activated Nrf2, inhibited nuclear localization of NF-κB, enhanced p62 expression and promoted autophagy in ethanol-induced mice, which in turn contributed to a positive feedback in Nrf2 activation (Qiu et al., 2017). It proposed a novel therapeutic option for chronic alcohol exposure induced liver disease.

One latest study found that DMY supplement also effectively ameliorated the development of NAFLD by suppressing hepatic lipid accumulation both in mice with HFD and in hepatocytes with PA-stimulation. They revealed that DMY restored the capacity of mitochondrial respiratory to attenuate oxidative stress. DMY increased SIRT3 expression by activating AMPK/PGC-1α/estrogen-related receptor-α (ERRα) signaling pathway. Interestingly, the benefits of DMY were unavailable in SIRT3 deficient mice and in hepatocytes with SIRT3 siRNA transfection or specific SIRT3 inhibitor pre-administration (Zeng X. et al., 2018). These results suggested new DMY-based preventive and therapeutic strategies for NAFLD.

Carbon tetrachloride (CCl_4_) was usually applied to induce chemical liver injury characterized by hepatocyte necrosis and liver dysfunction, and high-dosage of CCl_4_ even led to ALF (Luo et al., 2017). Previous studies have demonstrated that DMY (150 mg/kg) treatment for 4 days after CCl_4_ administration promoted hepatocyte proliferation and reduced necrosis or apoptosis. DMY exerted powerful anti-inflammatory effect and anti-oxidative effect with significant decrease on serum aspartate aminotransferase (AST), ALT, IL-1β, IL-6 levels but enhancement on SOD activity. Furthermore, c-Jun N-terminal kinase (JNK) inhibitor SP600125 attenuated TNF-α expression decrease and survival rate enhancement of ALF after DMY treatment, which suggested that DMY alleviated CCl_4_-induced acute liver injury via a JNK-dependent manner (Xie et al., 2015). DMY also prevented oxidative stress in the liver of LDL receptor deficient (LDLr^−/−^) mice with HFD, which might be related to normalizing antioxidant enzymes’ activities to suppress ROS generation and NOX2 expression (Liu et al., 2017). These results suggested that anti-oxidative ability was possibly vital mechanisms for the hepatoprotective effects of DMY.

Dihydromyricetin administration in male Sprague-Dawley (SD) rats with a HFD (60% fat) supplement reduced hepatic insulin resistance and alleviated steatosis in the liver via AMPK phosphorylation. DMY also ameliorated phosphorylation of Akt2 at Ser474 and IRS-1 at Ser612, decreased GSK-3β phosphorylation, and down-regulated glucose-6-phosphatase (G6Pase) and phosphoenolpyruvate caroxykinase (PEPCK) expression in HepG2 cells with high glucose stimulation (Le et al., 2016). It is suggested that DMY might be developed as a novel agent to improve hepatic insulin resistance in diabetes.

Nowadays, there are some clinical trials aiming to investigate the effect of DMY on NAFLD. In a double-blind clinical trial, two DMY capsules (150 mg) twice daily for 3 months significantly decreased alanine, AST, γ-glutamyl transpeptidase (GGT), glucose, LDL-cholesterol, apolipoprotein B (apoB), and the homeostasis model assessment of insulin resistance (HOMA-IR) index in adult patients suffering from NAFLD. DMY also evidently decreased the levels of TNF-α, cytokeratin-18 fragment (CK-18) and fibroblast growth factor 21 (FGF-21) but increased adiponectin level in the serum (Chen et al., 2015). This trial provides sufficient basis for the application of DMY in clinic.

## Neuroprotection

Growing evidence has shown that up-regulation of microRNAs (miRNAs) are involved in aging-related diseases. D-galactose (D-gal) significantly increased miR-34a in the brain aging model of rats, which was restored by DMY treatment with dosages of 100 and 200 mg/kg/d. Moreover, DMY improved learning and memory impairment, and mitigated neurons aging in D-gal-induced brain aging model of rats. DMY significantly inhibited apoptosis and rescued dysfunctional autophagy of neurons in hippocampus of D-gal-induced brain aging model of rats through sirtuin 1 (SIRT1) up-regulation and p53/p21 as well as mammalian target of rapamycin (mTOR) down-regulation via a miR-34a dependent manner (Kou et al., 2016). So far as we know, Alzheimer disease (AD), a degenerative disease of the central nervous system, is mainly manifested as devastating neurodegeneration and cognitive dysfunction (Jin et al., 2018). Associated with above results, DMY possibly has the potential to attenuate AD.

Parkinson’s disease (PD) is also called paralysis agitans or shaking palsy, and is characterized by gradual depletion of dopamine (DA) due to the selective loss of dopaminergic neurons. Catechol *O*-methyltransferase (COMT) is an enzyme participated in the metabolism of levodopa (L-dopa), and inhibition of COMT activity significantly increased the bioavailability of L-dopa. Human liver cytosol-catalyzed L-dopa methylation reaction *in vitro* confirmed that DMY successfully inhibited COMT activity in a dose-dependent manner (Zhu and Jia, 2014). PD can be induced by dopaminergic neurotoxic compound1-methyl-4-phenyl-1,2,3,6-tetrahydropyridine (MTPT) in the lab. DMY obviously restored the behavioral impairments, protected DA neurons and reduced ROS production after MPTP stimulation. Moreover, the inhibitory effect on GSK-3β activity possibly contributed to DA neuronal protection (Ren et al., 2016). These data revealed that DMY might be an ideal candidate for PD treatment.

Major depressive disorder (MDD) is a recurrent and highly prevalent disease. A growing body of evidence suggested that brain derived neurotrophic factor (BDNF) and neuroinflammation have a close association with depression-like behavior (Chan et al., 2017). There was less BDNF expression in the hippocampus of patients with MDD (Nuernberg et al., 2016). In addition, neuroinflammation and some inflammatory mediators also constitute biomarkers in the development of MDD. One latest report confirmed that DMY (10 and 20 mg/kg) pre-treatment for 3 days but not 1 or 2 days reduced immobility time in the tail suspension test (TST) and forced swimming test (FST). DMY also decreased immobility time of TST and FST, suppressed expressions of TNF-α and IL-6 mRNA, but enhanced locomotion of total distance traveled in mice after acute lipopolysaccharide (LPS) injection. DMY treatment for 7 days increased in sucrose preference but decreased FST and TST time in chronic unpredictable mild stress (CUMS) model. The attenuation effects on CUMS-induced depression-related and anxiety-related behaviors were attributed to the elevated BDNF pathway (Ren et al., 2018). These findings provide the solid evidence that DMY may be a promising candidate for the treatment of MDD.

Hypoxia may cause hippocampal neurodegeneration and lead to memory impairment. Adult male rats were subjected to hypobaric hypoxia (HH) for 7 days and the possible effect of DMY for HH rats was explored. The results showed that DMY pre-treatment preserved memory, improved synapses structures of hippocampal neurons, promoted mitochondrial biogenesis, decreased lipid peroxidation and suppressed ROS generation in HH-stimulated rats. Further experiments found that DMY increased SIRT3 expression and activity to induce FOXO3 deacetylation and protect against oxidative stress in hippocampal neurons and HT22 cells with hypoxia exposure (Liu P. et al., 2016). The findings suggest new strategies for preventing neurodegeneration and enhancing cognitive function after exposure to hypoxia.

Fetal alcohol exposure (FAE) includes a range of fetal diseases with behavioral and physiological disturbances. To our knowledge, gamma-aminobutyric acid (GABA) is an important inhibitory transmitter in central nervous system, and GABA_A_ receptor (GABA_A_R) is one of pharmacological targets of alcohol. DMY could selectively antagonize ethanol (EtOH)’s effects both *in vivo* and *in vitro* and enhance GABA_A_R function (Liang et al., 2014). Previous studies also found that DMY could counteract the effects of alcohol intoxication in adult rats (Shen et al., 2012). Recently, the researchers have verified that DMY administration increased entry time, loss of righting reflex (LORR), a duration and onset time of pentylenetetrazol (PTZ)-induced seizures, but decreased PTZ-induced seizures duration. DMY also prevented FAE-induced birth and growth deficits. Moreover, DMY prevented FAE-induced changes in responsiveness of synaptic and extrasynaptic GABA_A_Rs to a selective GABA_A_R agonist (Liang et al., 2014). It is noted that DMY alone exhibited no adverse effect on litter size, progeny weight, anxiety level and behavior in pregnant rats. The protective ability of DMY on FAE consequences without adverse side effects suggests that DMY is an attractive candidate for development as a treatment for prevention of fetal alcohol spectrum disorders.

Accumulation of methylglyoxal (MG), an endogenous toxic compound, may induce cognitive dysfunction. MG could enhance oxidative stress and calcium overload to result in mitochondrial apoptosis in PC12 cells. Evidence showed that DMY was able to improve glucose metabolism, suppress oxidative stress and inhibit apoptosis via the AMPK/glucose transporter 4 (GLUT4) signaling pathway in MG-stimulated PC12 cells (Jiang et al., 2014). In another study, DMY also dose-dependently protected sodium nitroprusside (SNP)-induced injury in PC12 cells via Akt and ERK1/2 signaling (Liao S.F. et al., 2014). 3-Nitropropionic acid (3-NP) also induced learning and memory impairments due to excessive ROS production. Research showed that DMY pre-treatment (10 mg/kg/day) decreased the time of initiating movement and passing across the beam, hang time, time to find the platform, suggesting that motor behavior, learning and memory ability was improved by DMY in 3-NP induced rats. The researchers found that DMY increased antioxidant capacity by increasing SOD activity to reduce MDA level. In addition, DMY up-regulated Bcl-2, but down-regulated cleaved caspase-3 and Bax to inhibit apoptosis (Mu et al., 2016). Above data indicated that the protective effect of DMY on behavioral deficits was mainly ascribed by the anti-oxidant and anti-apoptosis mechanism.

## Anti-Tumor

Hepatocellular carcinoma (HCC) is a disease with a high mortality rate because of the late diagnosis. Studies have shown that DMY could restrain the growth of HCC cells and activate apoptosis in hepatocarcinoma HepG2 cells. The molecular mechanism were attributed to inhibition of Akt-Ser473 phosphorylation, promotion of Bax and Bcl-2-associated death promoter (Bad) expression, as well as suppression of Bad-Ser112/Ser136 phosphorylation. DMY also suppressed Bcl-2 expression and enhanced the cleavage and activation of caspase-3. These data suggested that DMY induced mitochondria-mediated apoptosis in HepG2 cells via Akt/Bad pathway down-regulation (Zhang et al., 2017). Besides, another group reported that DMY altered the expression of cell cycle proteins such as checkpoint kinase (Chk), cyclin-dependent kinase 1 (Cdk1), cyclin A, cyclin B1, cell division cycle (Cdc) 25c, p-Cdc25c and p53, which suggested that DMY induced G2/M arrest. Moreover, Chk2 siRNA, but not p53 or Chk1, abolished G2/M arrest by DMY (Huang et al., 2013). In addition, one study confirmed that DMY significantly induced autophagy to inhibit cell proliferation in HepG2 cells. Further study indicated that DMY suppressed mTOR activation via regulating ERK1/2, AMPK, and PI3K/Akt (Xia et al., 2014). DMY also inhibited cell viability and promoted apoptosis in mouse hepatocellular carcinoma Hepal-6 cells, which was associated with ROS production down-regulation via decreasing transforming growth factor β (TGF-β), Smad3 and nicotinamide adenine dinucleotide phosphate oxidase 4 (NOX4) expression (Liu et al., 2015). Moreover, DMY also strongly inhibited the invasion and migration of the two different hepatoma cell lines human liver adenocarcinoma cells SK-Hep-1 and human hepatocellular carcinoma MHCC97. Further studies suggested that DMY up-regulated protein kinase C (PKC)-δ to decrease MMP-9 expression via inhibiting phosphorylation of p38, ERK1/2 and JNK (Zhang et al., 2014). Altogether, DMY may be a promising therapeutic medication for hepatocellular carcinoma growth and metastasis.

In human NSCLC, studies revealed that DMY exerted a selective cytotoxic to A549 and H1975 NSCLC cells, but not normal WI-38 fibroblasts cells. DMY induced cell apoptosis by depolarization of mitochondrial membrane. Moreover, DMY increased intracellular peroxide and sustained the activation of ERK1/2 and JNK1/2 signaling pathways, which was reversed by ROS scavenger *N*-acetylcysteine (NAC) (Kao et al., 2017). Furthermore, the study indicated that suppression of ROS-mediated ERK and JNK activation sensitized DMY-induced mitochondrial apoptosis in NSCLC. Therefore, combined treatment of DMY with ERK or JNK inhibitors might be ideal strategies for NSCLC.

Osteosarcoma is the most common primary malignant bone tumor in childhood and adolescence. DMY increased p21 expression and induced G2-M cell cycle arrest to cause DNA damage in osteosarcoma cells. Mechanistic analysis showed that the anti-tumor potential of DMY may be attributed to GSK3β inactivation via AMPKα and p38 activation (Zhao et al., 2014). Another study investigated the effect of DMY on hydrogen peroxide induced oxidative stress in the osteosarcoma cells. It was found that DMY prevented hydrogen peroxide induced viability reduction and apoptosis induction in MG63 cells through caspase inhibition and Bcl-2 activation (Wang et al., 2017). These similar data indicated that DMY may be a therapeutic candidate for the treatment of osteosarcoma.

All-*trans* retinoic acid (ATRA) is an intermediate product of vitamin A metabolism with a wide range of physiological and pharmacological activities. It is successful for patients with acute promyelocytic leukemia (APL). One study found that there was a strong synergy to promote APL NB4 cell differentiation with combined treatment of ATRA and DMY. And the enhanced differentiation was dependent on p38-STAT1 activation signaling pathway (He et al., 2018). It suggested that joint use of DMY and ATRA had synergistic effect on NB4 cell differentiation, which proposed new opportunities for the combination of DMY and ATRA as a promising approach for future differentiation therapy.

Adriamycin, exerting antitumor effects by inserting cellular DNA, has been challenged by the cardiotoxicity. Intriguingly, DMY not only prevented cardiotoxicity but also enhanced anticancer activity in human leukemia U937 cells and xenograft models (Zhu et al., 2015). Above data indicated that DMY might be used to potentiate anticancer activities and to increase the therapeutic window of adriamycin.

Ovarian cancer is one of the most common tumors in female genital organs with the highest fatality rate in gynecological cancer. One study demonstrated that DMY treatment induced G0/G1 and S phase arrest in ovarian cancer cells. DMY also effectively induced cell apoptosis by p53-mediated survivin down-regulation. Moreover, DMY markedly reversed paclitaxel (PTX) and doxorubicin (DOX) resistance against ovarian cancer cells (Xu Y. et al., 2017). It is suggested that DMY might be a candidate chemotherapeutic agent for the treatment of ovarian cancer.

Dihydromyricetin also inhibited proliferation, induced cell cytotoxicity and promoted apoptosis by p53 up-regulation and Bcl-2 down-regulation in AGS human gastric cancer cells (Ji et al., 2015). It is strongly suggested that DMY might be a potential therapeutic compound for gastric cancer treatment.

## Dermatoprotection

One group found that DMY effectively inhibited tyrosinase activity and reduced melanin amount in cells. The mechanism might be related to the inhibition of oxidative stress, down-regulation of protein kinase A (PKA), PKC and mitogen-activated protein kinase (MAPK) signaling pathways (Huang et al., 2016). The study indicated that DMY had the potential to be developed as a depigmentation skin care product.

Despite a relatively low incidence of melanoma, metastatic melanoma is responsible for about 80% of deaths caused by skin cancer. Previous studies demonstrated that DMY suppressed cell proliferation, induced cell cycle arrest at G1/S phase and promoted apoptosis of melanoma SK-MEL-28 cells, which might be related to the enhanced expression of Bax proteins and decreased levels of IKK-α, NF-κB, and P-p38 (Zeng et al., 2014). Additionally, DMY induced apoptosis and cytoprotective autophagy through ROS-NF-κB signaling in human melanoma cells (Zhou et al., 2017). All these results suggested that DMY may be a novel and effective agent to attenuate growth of melanoma.

Another group found that DMY (1.25–10 μM) pre-administration increased ultraviolet A (UVA)-induced cell viability but suppressed UVA-induced inflammation, ROS production and apoptosis in the human keratinocyte cell line HaCaT cells. DMY increased mitochondrial membrane potential, Bcl-2 and Bcl-xl expression while decreased Bax level and caspase activation. Moreover, DMY blocked NF-κB/p65 translocation into nucleus and inhibited JNK phosphorylation (He et al., 2016). Therefore, DHM may be potentially developed to protect against UVA-induced skin damage.

## Other Effects

*Staphylococcus aureus* (*S. aureus*) is a common pathogenic bacteria, which produces many toxins and invasive enzymes *in vivo*. *S. aureus* may cause local pyogenic infection and systemic infections such as pneumonia, pseudomembranous colitis, pericarditis, and even septicemia. It was found that DMY treatment destroyed the cell membrane integrity of *S. aureus*. Meanwhile, DMY significantly reduced membrane fluidity and altered the conformation of membrane protein, which was probably due to interaction with membrane lipids and proteins. Moreover, DMY achieved bactericidal activity by interacting with intracellular DNA through a groove binding mode in *S. aureus* (Wu Y. et al., 2017). In general, these results suggested that DMY has great value to be developed as a new food preservative.

Osteoporosis is a metabolic bone lesion characterized by low bone mass and structural damage of bone tissue. Research has shown that DMY inhibited osteoclast differentiation and activation, attenuated bone resorption function, reduced osteoclast actin ring formation *in vitro*. Further studies indicated that DMY inhibited osteoclastogenesis through multiple pathways. DMY decreased the ratio of receptor activator for nuclear factor-κB ligand (RANKL) to osteoprotegerin in serum and inhibited several inflammatory cytokines generation. DMY also suppressed multiple pathways downstream of RANKL signaling, such as MAPKs, ROS, PI3K/Akt, NF-κB, and activator protein 1 (AP-1) in osteoclast precursor-like cell line and osteoclasts (Zhao et al., 2017a). These data suggested that DMY might be used for the treatment of osteoclast-related diseases such as rheumatoid arthritis and osteoporosis.

Asthma is a chronic inflammatory disease of the airways mediated by a variety of cells (such as eosinophils, macrophages, lymphocytes, neutrophils, airway epithelial cells, etc.) and cellular components. DMY obviously down-regulated the levels of IL-4, IL-5, IL-13 and reduced the amount of inflammatory cells in the bronchoalveolar lavage (BAL) fluid from ovalbumin (OVA) induced asthma of mice (Xu B. et al., 2017). It is suggested that DMY was possible a potential anti-inflammatory agent for asthma.

In the kidney tissues, aggregation of calcium induces kidney stones formation and kidney injury. Experimental results showed that DMY decreased the level of kidney injury molecule-1 and blood urea nitrogen (BUN) as well as calcium in LPS-induced acute kidney injury (AKI) of rats. DMY inhibited the aggregation of calcium oxalate crystals and decreased the level of Ca/creatinine in the urine. The levels of CD44 and osteopontin and the population of TUNEL-positive cells in the kidney were obviously decreased after DMY treatment. In addition, DMY also exerted anti-oxidative effect in the kidney. In summary, DMY prevented endotoxemia-induced AKI by reducing calcium aggregation and inhibiting ROS generation (Wang et al., 2016). Cisplatin is a major antineoplastic drug with severe side effects. High-dose cisplatin is likely to induce nephrotoxicity or even permanent reduction of kidney function. DMY treatment significantly decreased the levels of BUN and serum creatinine (SCr), ameliorated structural damage and kidney functional impairment induced by cisplatin (Wu et al., 2016). Thus, DMY may be beneficial for the treatment of AKI.

Transplantation of bone marrow-derived mesenchymal stem cell (bmMSC) is a promising new strategy for the treatment of several diseases. Studies found that DMY enhanced mesenchymal stem cells viability with ⋅ OH-stimulation, which may be attributed to antioxidant effect of DMY. Direct scavenging of free radical and Fe^2+^-chelation might be involved in the detailed mechanisms (Li et al., 2016). This protective effect indicates that DMY may be a promising agent for cell transplantation therapy.

## Concluding Remarks and Future Perpectives

The bioavailability of polyphenolic compound is influenced by their transmembrane capacity and their structure. Many experiments found that polyphenolics had low oral bioavailability (Teng and Chen, 2018). Flavonoids widely exist in our daily diet, such as fruits, cocoa, and tea (Chen et al., 2018b). In food, flavonoids most exist in glycosylated forms, and glycosylation could influence the absorption (Chen et al., 2018a).

The DMY is a flavonoid in *A. grossedentata*, which is also a polyphenolic compound. Poor stability, low solubility, rapid metabolism and absorption usually lead to weak bioavailability and inefficient drug-ability of DMY, which limits its potential medicinal applications. And there are still several specific mechanisms that have not been identified well. However, DMY possessed various pharmacological activities and exerts many different kinds of benefits especially in the cardiovascular system, liver, nervous system, skin system as well as the improvement effect in metabolic disease such as diabetes. A large number of studies confirmed that DMY exert protective or preventive effect through anti-oxidation, anti-inflammation, anti-apoptosis and other pathway. And the detailed mechanisms may be associated with several different molecules involved in cellular apoptosis, oxidative stress, and inflammation, such as AMPK, MAPK, Akt, NF-κB, Nrf2, ABCA1, PPARγ and so on (Supplementary Table S1).

Anyway, from the point of view of pharmacology, DMY is a promising flavonoid compound for preventing initiation and progression of several diseases. Despite the reported hypothesized mechanisms, further exhaustive mechanistic and toxicological studies on DMY are necessary to accelerate experimental research or preclinical study into commercial drugs in the near future.

## Author Contributions

JZ, YC, GM, and SY designed this work and figures, collected and analyzed the data, co-wrote the manuscript, and edited the manuscript. HL, LS, MX, JY, and QZ gathered the data.

## Conflict of Interest Statement

The authors declare that the research was conducted in the absence of any commercial or financial relationships that could be construed as a potential conflict of interest.
