# Supplementary material for: Recent Update on the Pharmacological Effects and Mechanisms of Dihydromyricetin
Source: Front Pharmacol. 2018 Oct 25;9:1204. doi: 10.3389/fphar.2018.01204 (PMC6209623; doi:10.3389/fphar.2018.01204)
Supplement: Supplementary file 1 [file Data_Sheet_1.PDF]

Table1 Main effects and possible mechanisms of DMY

	pathological state	Objects	Main Effects	Possible Mechanisms
**cardio-vascular system**	atherosclerosis	PA-stimulated HUVECs	cell viability↑, inflammation↓	Nrf2 signal pathway↑, ROS↓, pyroptosis↓
		ox-LDL-stimulated macrophages	lipid accumulation↓	ABCA1↑, ABCG1↑, SR-B1↑
		$LDLr^{-/-}$ mice with HFD	inflammation↓, cholesterol efflux↑	NOX2↓, NF-κB↓, ICAM-1↓, VCAM-1↓, ROS↓
		TNF-α- stimulated HeLa cells	inflammation↓	IKK↓, p-IκBα↓, TRAF2↓, RIP1↓, NF-κB↓
	I/R	I/R in H9c2 cells<br>myocardial IR in rats	I/R injury↓	antioxidant capacity↑, PI3K↑, Akt↑, HIF-1α↓, apoptosis↓
	cardiotoxicity	ADR administrated mice	survival rate↑, cardiotoxicity↓	apoptosis↓, ROS↓, MDM2↓
	arrhythmias	aconitine administrated rats	arrhythmias incidence↓	I_{K1}↑, I_{Na}↓
	myocardial remodeling	Ang II stimulated cardiomyocyte	cardiomyocyte hypertrophy↓	cGMP↑, p-eNOS↑
		Ang II stimulated cardiac fibroblasts	fibroblasts proliferation↓	Trx↑, SOD↑, $p22^{phox}$↓, ROS↓
		TAC in mice	myocardial hypertrophy↓	SIRT3↑, ROS↓
	PAH	MCT induced PAH in rats	pulmonary vasculature remodeling↓	p-STAT3↓, MMP9↓
		IL-6-stimulated HPASMCs	cell migration↓	p-STAT3↓, MMP9↓
		H_2O_2-stimulated HUVECs	cell injury↓	Bcl-2↑, ROS↓, p53↓, PARP cleavage↓

**diabetes**	DM	HFD fed rats	insulin resistance↓	p-AKT↑, p-AMPK↑, p-GSK-3β↓
		differentiated C2C12 myotubes	insulin sensitivity↑	p-AMPK↑, PGC-1α↑, SIRT3↑
		dexamethasone-treated adipocytes	glucose uptake↑, adipogenesis↓	p-PPARγ↓, p-ERK↓, CDK5↓
		STZ-administrated mice	diabetic cardiomyopathy↓	ATP↑, p-AMPK↑, p-ULK1↑, GSH-Px↑
**liver**	I/R	liver I/R in mice	I/R injury↓	Atg5↑, Atg12↑, beclin1↑, LC3↑, FOXO3a↑
	ALD	ethanol administrated mice	hepatic lipid peroxidation↓	GSH↑, Nrf2↑, p62↑, NF-κB↓
	NAFLD	HFD fed mice	hepatic lipid accumulation↓	AMPK/ PGC-1α/ERRα↑, SIRT3↑, ROS↓
		PA-stimulated hepatocytes		
		patients suffering from NAFLD	liver function↑, insulin resistance↓	TNF-α↓, CK-18↓, FGF-21↓
	ALF	CCl_4 administrated mice	hepatocyte proliferation↑	SOD↑, IL-1β↓, IL-6↓, ROS↓
**nervous system**	AD	D-gal induced brain aging models	neurons aging↓	SIRT1↑, miR-34a↓, p53/p21↓, mTOR↓
	PD	MTPT administrated mice	DA neurons↑	COMT↓, ROS↓, GSK-3β↓
	MDD	CUMS models	depression-related behaviors↓	BDNF↑
	hypoxia injury	HH induced rats	memory↑, synapses structures↑	SIRT3↑, FOXO3 deacetylation↑, ROS↓
	FAE	etOH administrated rats	alcohol intoxication↓	$GABA_AR$ function↑
	memory impairments	3-NP induced rats	memory ability↑	Bcl-2↑, SOD↑, ROS↓, cleaved caspase-3↓, Bax↓

**tumor**	HCC	HepG2 cells, Hepal-6 cells, SK-Hep-1 cells	cell apoptosis↑	Bax↑, Bad↑, caspase-3↑, PKC-δ↑, Bcl-2↓, mTOR↓
	NSCLC	A549 and H1975 cells	cell apoptosis↑	ERK1/2↑, JNK↑, ROS↑
	osteosarcoma	MG63 cells	DNA damage↑	p21↑, AMPKα↑, p-38↑, GSK3β↓
	APL	NB4 cells	cell differentiation↑	p38-STAT1↑
	ovarian cancer	A2780 and SKOV3 cells	cell apoptosis↑	p53-mediated survivin↓
**skin**	skin depigmenting	B16F10 mouse melanoma cells	tyrosinase activity↓, melanin amount↓	ROS↓, PKA↓, PKC↓, MAPK↓
	melanoma	SK-MEL-28 cells	cell proliferation↓, cell apoptosis↑	G1/S arrest↑, Bax↑, IKK-α↓, NF-κB↓, p-p38↓
	UVA-induced skin damage	UVA-stimulated HaCaT cells	cell viability↑, inflammation↓	Bcl-2↑, Bax↓, caspase↓, NF-κB/p65↓, p- JNK↓
